# PMP2 regulates myelin thickening and ATP production during remyelination

**DOI:** 10.1002/glia.24508

**Published:** 2024-02-05

**Authors:** Jiayue Hong, Rebekah Garfolo, Sejal Kabre, Christian Humml, Viktorija Velanac, Clémence Roué, Brianna Beck, Haley Jeanette, Sarah Haslam, Martin Bach, Simar Arora, Jenica Acheta, Klaus-Armin Nave, Markus H. Schwab, David Jourd’heuil, Yannick Poitelon, Sophie Belin

**Affiliations:** 1 Department of Neuroscience and Experimental Therapeutics, Albany Medical College, Albany, New York, USA; 2 Department of Neurogenetics, Max Planck Institute for Multidisciplinary Sciences, Göttingen, Germany; 3 Paul Flechsig Institute of Neuropathology, University Hospital Leipzig, Leipzig, Germany; 4 Department of Molecular and Cellular Physiology, Albany Medical College, Albany, New York, USA

**Keywords:** FABP8, myelin, NRG1t3, PMP2, Schwann cell

## Abstract

It is well established that axonal Neuregulin 1 type 3 (NRG1t3) regulates developmental myelin formation as well as EGR2-dependent gene activation and lipid synthesis. However, in peripheral neuropathy disease context, elevated axonal NRG1t3 improves remyelination and myelin sheath thickness without increasing *Egr2* expression or activity, and without affecting the transcriptional activity of canonical myelination genes. Surprisingly, *Pmp2*, encoding for a myelin fatty acid binding protein, is the only gene whose expression increases in Schwann cells following overexpression of axonal NRG1t3. Here, we demonstrate PMP2 expression is directly regulated by NRG1t3 active form, following proteolytic cleavage. Then, using a transgenic mouse model overexpressing axonal NRG1t3 (NRG1t3OE) and knocked out for PMP2, we demonstrate that PMP2 is required for NRG1t3-mediated remyelination. We demonstrate that the sustained expression of *Pmp2* in NRG1t3OE mice enhances the fatty acid uptake in sciatic nerve fibers and the mitochondrial ATP production in Schwann cells. In sum, our findings demonstrate that PMP2 is a direct downstream mediator of NRG1t3 and that the modulation of PMP2 downstream NRG1t3 activation has distinct effects on Schwann cell function during developmental myelination and remyelination.

## Graphical Abstract

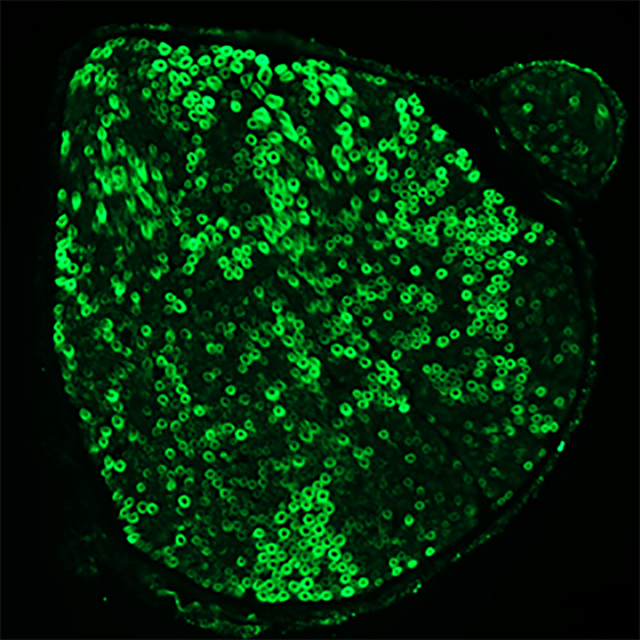

Immunostaining of PMP2 in the sciatic nerve of a mouse

## 1 | INTRODUCTION

In health and disease, the capacity of Schwann cells to produce myelin is regulated by NRG1t3 (Michailov et al., 2004; Nave & Salzer, 2006; Taveggia et al., 2005). NRG1t3 is an axonal transmembrane protein, which is proteolytically activated by the BACE1 protease (Velanac et al., 2012; Willem et al., 2006). The activated form of axonal NRG1t3 binds to Schwann cell ErbB2/ErbB3 receptors which activate downstream signal transduction pathways in Schwann cells (e.g., PI3K-AKT and MAPK/ERK) (Pereira et al., 2012; Taveggia et al., 2005; Taveggia et al., 2010). These pathways activate the transcriptional master regulator EGR2 (Kao et al., 2009; Parkinson et al., 2004). During development, axonal NRG1t3 is required for Schwann cell proliferation, differentiation and controls the myelinating fate of axons (Michailov et al., 2004; Taveggia et al., 2005; Wolpowitz et al., 2000). After an injury, NRG1t3 supports nerve regeneration and Schwann cell remyelination (Fricker et al., 2011; Fricker et al., 2013). In addition, overexpression of the axonal NRG1t3 promotes myelin formation during development (Michailov et al., 2004; Taveggia et al., 2005), after injury (Stassart et al., 2013), or in animal models of inherited peripheral demyelinating neuropathies (Belin, Ornaghi, et al., 2019; Scapin et al., 2019).

It was determined, only recently, that overexpression of NRG1t3 triggers a non-canonical signaling pathway, independent of EGR2, favoring the enrichment of myelin lipids over myelin proteins (Belin, Ornaghi, et al., 2019; Scapin et al., 2019). When axonal NRG1t3 expression is enhanced, the composition of the resulting thicker myelin is enriched in cholesterol, saturated, and unsaturated fatty acids (Scapin et al., 2019) in comparison to control nerves with normal levels of NRG1t3. In addition, rather than a global myelin protein up-regulation, PMP2, a fatty acid-binding protein, is uniquely up-regulated downstream NRG1t3 overexpression in Schwann cells (Belin, Ornaghi, et al., 2019; Scapin et al., 2019).

PMP2 (also known as FABP8) is a unique small *β*-barrel cytosolic myelin protein carrying long-chain fatty acid inside its *β*-barrel structure. Under normal conditions, PMP2 presents a unique mosaic expression, limited to a subset of Schwann cells ensheathing large motor neuron axons and even more intriguing to a limited subset of Schwann cells along the same axon (Yim et al., 2022; Zenker et al., 2014). PMP2-positive Schwann cells have a thicker myelin than PMP2-negative Schwann cells (Yim et al., 2022). Since PMP2 can bind to lipid bilayers via two opposing faces, sticking membranes together with a constant spacing (Ruskamo et al., 2014; Sedzik et al., 1985), it was thought to function in myelin assembly, stabilization, or turnover. However, mice ablated for *Pmp2* have a transient mild hypomyelination and do not develop any abnormal myelin compaction during development, in adulthood, or after injury (Stettner et al., 2018; Zenker et al., 2014). In addition, CMT1G mutants (mutated in *Pmp2*) revealed a similar capacity to bind membrane bilayers, which indicates that the surface properties of PMP2 in the CMT1G mutants are unaltered (Ruskamo et al., 2017; Uusitalo et al., 2021). The peak of PMP2 expression is restricted to early development in mice (post-natal day 10) (Gerber et al., 2021; Zenker et al., 2014), a timeframe during which PMP2 absence transiently alters the composition of myelin lipids (Zenker et al., 2014). There is evidence that loss of PMP2 also transiently alters myelination and nerve function during early remyelination in mice (from 7 to 14 days post-crush injury) (Stettner et al., 2018). However, despite the research conducted on PMP2 using loss of function approaches in Schwann cells (Stettner et al., 2018; Zenker et al., 2014), the molecular mechanisms by which PMP2 regulates myelin lipid metabolism during Schwann cell development and remyelination are unknown. What regulates PMP2 level of expression in Schwann cells is still unknown and the role of PMP2 as a fatty acid chaperone is largely understudied.

Intriguingly, both sustained activation of axonal NRG1t3 or MAPK/ERK increases myelin formation and PMP2 protein (Belin, Ornaghi, et al., 2019; Sheean et al., 2014). While PMP2 is the only myelin protein with a sustained elevated expression after NRG1t3 enhanced expression (~4 fold, up to 1 year of age) (Belin, Ornaghi, et al., 2019), whether PMP2 is necessary to promote myelin formation downstream NRG1t3/MAPK/ERK sustained activation is unknown.

In this study, we demonstrate that *Pmp2* expression is regulated in part by BACE1-mediated NRG1t3 processing in mouse sciatic nerves. To clarify the function of PMP2 upregulation downstream of NRG1t3-mediated hypermyelination during development and remyelination, we generated mice overexpressing NRG1t3 and knocked out for *Pmp2*. We found that while the loss of PMP2 does not alter NRG1t3-mediated hypermyelination during development, it impedes the increase in myelin thickness mediated by NRG1t3 overexpression after nerve crush injury. Finally, we demonstrated a new role for PMP2 in modulating Schwann cell energy homeostasis, ATP production, and fatty acid uptake.

## 2 | METHODS

### 2.1 | Transgenic mice

All experiments involving mice followed experimental protocols approved by the Albany Medical College Institutional Animal Care and Use Committee. *N*-terminally hemagglutinin-tagged transgenic mice expressing either full-length NRG1 type III (hereafter called Nrg1t3OE) or a NRG1 type III variant mimicking BACE1 processing (NRG1t3ΔC) under control of neuronal promoter Thy1.2, *Bace1*^−/−^ were previously described (Taveggia et al., 2005; Velanac et al., 2012). *Pmp2*^−/−^ (C57BL/6N-Pmp2^tm1(KOMP)Vlcg^) mice were obtained from the MMRRC. The genotype of *Pmp2*^−/−^ mice was determined by PCR analysis with the *Pmp2* forward primers KOF-5′-CTAACCCCACACCT GAAAAGCAAGG-3′, WTF-5′-GCAGCCTCTGTTCCACATACACTTCA-3′ and the *Pmp2* reverse primer R-5′-GTCTGCCCAGAACAAAGCT CTTACC-3′ that amplified 200 bp and 400 bp nucleotide fragments for knockout and wildtype allele, respectively. PCR conditions were 94°C for 5 min, 30 cycles of 94°C for 15 s, and 61°C for 30 s, 72°C for 40 s, followed by 5 min extension at 72°C, in a standard PCR reaction mix. Mice were housed in cages of 5 in 12/12-h light/dark cycles. No mice were excluded from the study. Equal numbers of males and females were included in the study. This study was carried out in accordance with the principles of the Basel Declaration and recommendations of ARRIVE guidelines issued by the NC3Rs and approved by the Albany Medical College Institutional Animal Care and Use Committee (no. 20–08002).

### 2.2 | Nerve crush

Nerve crush was performed on adult mice using an aseptic technique under a laminar flow hood, as previously described (Jeanette et al., 2021). Mice were anesthetized with isoflurane and eyes were lubricated. While the mouse lay flat on its ventral side, the region of the intended crush was shaved and cleaned with iodine and ethanol. On one leg, a small incision was made in the skin and muscle above the sciatic nerve near the sciatic notch. Once the nerve was exposed, any membranous ties to the muscle were severed using small scissors. A pair of hemostatic forceps were dipped in liquid N_2_ for 2 s and dipped in activated carbon. Across mouse cohorts, the sciatic crush site was consistently done at 0.5 cm distal to the sciatic notch by tight compression with chilled hemostatic forceps for 60 s. The nerve was crushed, not severed. The other leg was unoperated. The injured skin was closed with a staple. Mice were monitored daily after surgery and analgesics were administered daily for 3 days post-surgery. For molecular or morphological analysis, 8 mm long injured nerves were consistently sampled at a 2 mm distance from the crush site.

### 2.3 | Electrophysiological analyses

Mutant and control littermates were analyzed as described previously (Acheta, Bhatia, et al., 2022). Mice were anesthetized with tribromoethanol 0.4 mg/g of body weight and placed under a heating lamp to avoid hypothermia. Motor action potential recordings of sciatic nerves were obtained with subdermal steel monopolar needle electrodes: a pair of stimulating electrodes were inserted subcutaneously near the nerve at the ankle, then at the sciatic notch, and finally at the paraspinal region at the level of the iliac crest to obtain three distinct sites of stimulation, proximal and distal, along the nerve. Electrophysiological studies comprising motor and sensory nerve conduction studies were conducted using a VikingQuest electromyography device. Measures were blindly evaluated during recordings.

### 2.4 | Morphological analysis

Sciatic nerves were dissected, as described previously (Acheta, Hong, et al., 2022). Nerves were fixed in 2% buffered glutaraldehyde and postfixed in 1% osmium tetroxide. After alcohol dehydration, the samples were embedded in Epon. Transverse sections (1 μm thick) were stained with toluidine blue and examined by light microscopy. For all morphological assessments, at least three mice per genotype were analyzed. For g ratio analysis of sciatic nerves (axon diameter/fiber diameter), semithin section images were acquired with *a* × 100 objective. G ratios were determined for at least 100 fibers chosen randomly per animal. Axon and fiber diameters, and the number of myelinated fibers were quantified on semithin sections using the ImageJ software (imagej.nih.gov/ij). Data were analyzed using GraphPad Prism 6.01. Images were blindly evaluated during analysis.

### 2.5 | Immunohistochemistry

For cross sections, OCT-embedded sciatic nerves were sliced into 10 μm-thick sections with cryostat and stored in −80C. Sections were thawed for 5 min, as previously described (Belin, Herron, et al., 2019). Nerve sections were permeabilized with −20C 100% methanol, washed in PBS, then incubated in blocking solution for 1 h at room temperature. Nerves were incubated overnight at 4C with primary Abs diluted in blocking solution. The following primary Abs were used: anti-rabbit PMP2 1/500 (ProteinTech, 12717–1-AP), anti-chicken NF-M 1/1000 (Biolegend, 822701). Nerves were washed, incubated with appropriate secondary Abs diluted in blocking solution for 1 h at room temperature, washed, stained with DAPI 1/10,000 for 5 min room temperature, washed, mounted with Vectashield, then sealed. Images were acquired at ×20 with a Zeiss epifluorescent microscope. Analysis was done using ImageJ Software (http://imagej.nih.gov/ij) (Colom et al., 2012). Images were blindly evaluated during analysis.

### 2.7 | BODIPY uptake

Sciatic nerves were sampled, the epineurium was carefully removed, and nerves fibers were gently teased apart. The teased fibers were placed in equilibration media (MEM, 10% Lipoprotein Deficient Fetal Bovine Serum) at 37C for 30 min, then incubated for 1 h in equilibration media supplemented with 10 μm BODIPY-C16. The teased fibers were washed with PBS, spread on a TESPA-coated slides and incubated with DAPI before to be mounted with Fluoromount-G Mounting Medium (ThermoFisher) for acquisition at ×20 with a Zeiss epifluorescent microscope. Analysis was done using ImageJ Software (http://imagej.nih.gov/ij) (Colom et al., 2012). Images were blindly evaluated during analysis.

### 2.7 | Western blot

Sciatic nerves were then frozen in liquid nitrogen, pulverized, and resuspended in lysis buffer (150 mm NaCl, 25 mm HEPES, 0.3% CHAPS, pH 7.4, 1 mm Na_3_VO_4_, 1 mm NaF and 1:100 Protease Inhibitor Cocktail [Roche Diagnostic, Florham Park, NJ]), as previously described (Della-Flora Nunes et al., 2017; Poitelon et al., 2018). Protein lysates were centrifuged at 15,000*g* for 30 min at 4C. Supernatant protein concentrations were determined by bicinchoninic acid assay protein assay (Thermo Scientific, Waltham, MA) according to the manufacturer’s instructions. Equal amounts of homogenates were diluted 3:1 in 4 Laemmli (250 mm Tris–HCl, pH 6.8, 8% sodium dodecyl sulfate, 8% β-mercaptoethanol, 40% glycerol, 0.02% bromophenol Blue), denatured 5 min at 100C, resolved on an SDS-polyacrylamide gel and electroblotted onto PVDF membrane. Blots were then blocked with 5% bovine serum albumin in 1× phosphate-buffered saline (PBS), 0.05% Tween-20, and incubated overnight with the following appropriate antibodies: anti-Calnexin 1/3000 (Sigma, C4731), anti-GAPDH 1/2000 (Sigma, G9545), anti-GAP43 1/2000 (Abcam, ab16053), anti-c-JUN 1/1000 (Cell Signaling, 60A8), anti-AKT (Cell Signaling, 9272), anti-p-AKT Ser473 (9271), anti-ERK 1/2 (Cell Signaling, 9102), anti-p-ERK 1/2 (Cell Signaling, 9102), anti-MBP 1/1000 (Biolegend, SMi-94 and 99P), anti-PMP2 1/500 (ProteinTech, 12717–1-AP), anti-P0 1/5000 (Aves Labs, PZO), anti-Tubulin (Proteintech 10094–1-AP), anti-Calnexin (Sigma, C4731), anti-GAPDH (Sigma, G9545). Membranes were then rinsed in 0.1% Tween in 1× PBS and incubated for 1 h with secondary antibodies. Blots were developed using ECL or ECL plus (GE Healthcare, Chicago, IL). Western blots were quantified using Image J software (http://imagej.nih.gov/ij).

### 2.8 | Mitochondrial function

A Seahorse XFe96 Analyzer instrument with the Mitochondrial Stress test kit was used according to the manufacturer’s instruction to dynamically measure the extracellular acidification rate (ECAR), a surrogate marker of glycolysis, and the oxygen consumption rate (OCR), a surrogate to basal oxygen consumption, as well as ATP-linked oxygen consumption and maximal respiration in the presence of a mitochondrial ATP synthase (oligomycin), and mitochondrial uncoupler (FCCP), respectively. Fresh 2 mm sciatic nerve segments of control or crush nerves at 7 days post injury (7 dpi) were cut and placed in the bottom of a PLL-coated XFe96 plate. Nerve sections were incubated in XF assay media for 1 h at 37°C. H^+^ production and O_2_ consumption were measured over 120 min and injected sequentially with oligomycin (1.5 μM) was administered to inhibit ATP synthase. FCCP (2.0 μM) to induce mitochondrial stress, and a combination of Rotenone (0.5 μM) and Antimycin A (0.5 μM) to shut down all mitochondrial activity. Data collected from sciatic nerve sections were normalized through protein quantification analysis, and are represented in pmoles/min/mg/ml. Formulas to calculate the extrapolations from H^+^ production and O_2_ consumption are as follows: Basal Respiration (minimal basal OCR−minimal Antimycin A/Rotenone OCR), Maximal Respiration (maximal FCCP OCR−minimal Antimycin A/Rotenone OCR), Mitochondrial ATP Production (2 × 2.75 × [minimal basal OCR − minimal Oligomycin OCR]), Glycolytic ATP production ([maximal basal ECAR × 2.28 × 1.6 × 2.5] − [basal respiration × 0.61]), Total ATP production (Mitochondrial ATP production + Glycolytic ATP Production).

### 2.9 | RNA preparation and real-time quantitative-PCR

Sciatic nerves were dissected, stripped of epineurium, frozen in liquid nitrogen, and pulverized. Total RNA was prepared from sciatic nerve or Schwann cells with TRIzol (Roche Diagnostic). One microgram of RNA was reverse transcribed using Superscript III (Invitrogen, Carlsbad, CA, United States) and processed as previously described (Belin, Ornaghi, et al., 2019). Shortly, for each reaction, 5 μM of oligo(dT)20 and 5 ng/μl random hexamers were used. Quantitative PCR was performed using 20 ng of cDNA combined with 1× FastStart Universal Probe Master (Roche Diagnostic). Data were analyzed using the threshold cycle (Ct) and 2(−ΔΔCt) method. GAPDH was used as control gene for PCR. Rps20 was used as endogenous gene of reference for the real time quantitative PCR (RTq-PCR) and Rps13 and Rpl27 were used as to validate the stable expression of Rps20. Primer sequences for FABPs were designed using Primer3 and are available in Table S1.

### 2.10 | Statistical analyses

Experiments were not randomized, but data collection and analysis were performed blind to the conditions of the experiments. Data are presented as mean ± standard error of the mean (S.E.M.). No statistical methods were used to predetermine sample sizes, but our sample sizes are similar to those generally employed in the field. Two-tailed Student’s *t* test or One-way ANOVA with Bonferroni correction was used for statistical analysis of the differences between multiple groups. Values of *p* value ≤0.05 were considered to represent a significant difference.

## 3 | RESULTS

### 3.1 | Glial PMP2 is regulated by axonal NRG1t3

**FIGURE 1:**
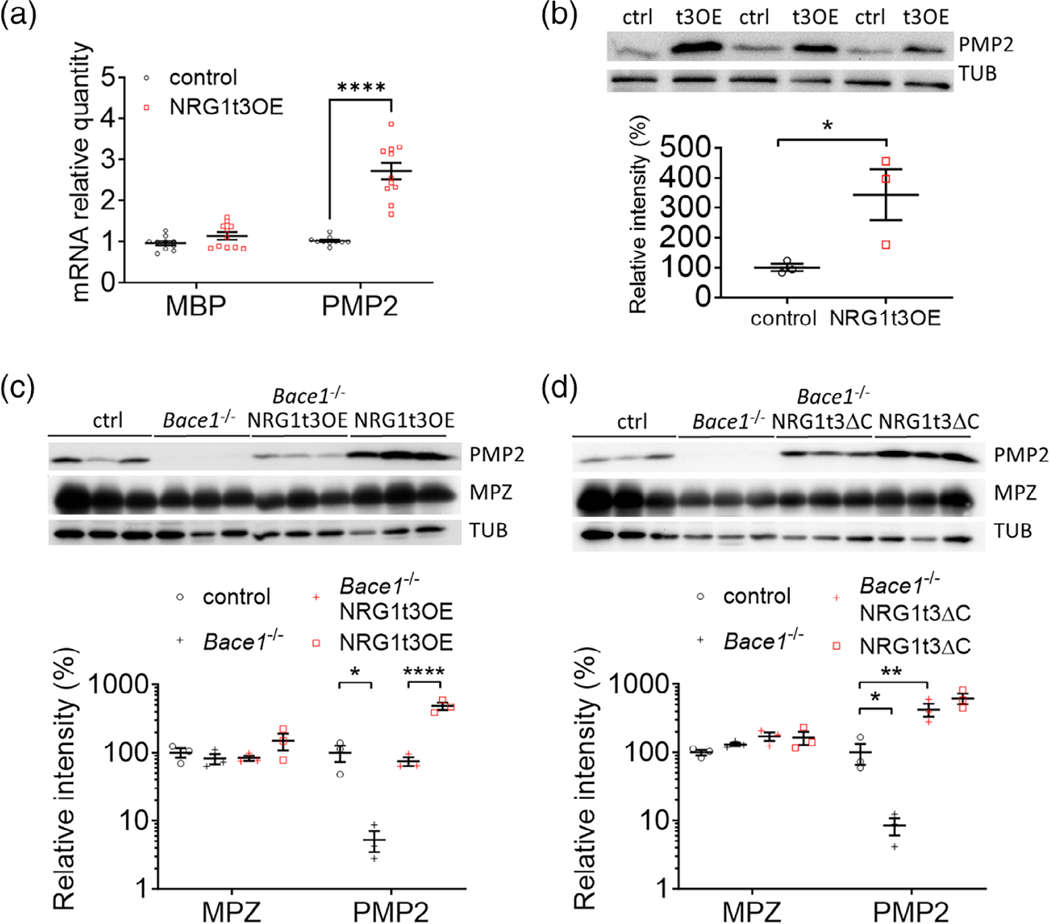
PMP2 is regulated by NRG1t3. (A) RTqPCR analysis of MBP and PMP2 in control and NRG1t3OE sciatic nerves at 7 days of age. (B–D) Western blot analysis of PMP2 and MPZ in control and NRG1t3OE sciatic nerves at 7 days of age (B), in control, *Bace1*^−/−^, NRG1t3OE, *Bace1*^−/−^; NRG1t3OE, NRG1t3ΔC (Constitutively active nNRG1 type III) and *Bace1*^−/−^; NRG1t3ΔC at 60 days of age (C,D). Tubulin (TUB) was used for the endogenous control for the Western blots. Error bars represent s.e.m., *n* = 3–11 mice, and each dot on the graph represents a different *n*. One-way ANOVA with Bonferroni correction (A,C,D) and two-tailed unpaired Student’s *t* test. (B). *p value <.05, ****p value <.0001. t3OE (NRG1t3OE).

We already established that *Pmp2* (also known as *Fabp8*) expression increases with the overexpression of the axonal NRG1t3 at 1 month of age (Belin, Ornaghi, et al., 2019; Scapin et al., 2019). Here we show that PMP2 is regulated similarly at postnatal day 7, which further supports PMP2 as a direct developmental target of NRG1t3 (Figure 1A,B). The processing of NRG1t3 by the secretase BACE1 was shown to positively regulate Schwann cell myelination and remyelination (Hu et al., 2008; Willem et al., 2006). To establish if PMP2 expression depends on the BACE1-dependent activation of NRG1t3, we took advantage of BACE1 full knockout (*Bace1*^−/−^) and transgenic mouse models overexpressing full length NRG1t3 (NRG1t3OE) or a NRG1t3 variant (NRG1t3ΔC also known as NRG1^GIEF^) which mimics the active form of NRG1t3 after BACE1 cleavage (Velanac et al., 2012). In absence of BACE1 expression, the activation of NRG1t3 (active cleaved form) is known to be significantly reduced (Willem et al., 2006). Here, we show that in *Bace1*^−/−^ mouse sciatic nerves PMP2 expression is almost undetectable (Figure 1C,D). When full length NRG1t3 was overexpressed in *Bace1*^−/−^ mice (*Bace1*^−/−^; NRG1t3OE), PMP2 was detected but significantly reduced compared to PMP2 expression level in NRG1t3OE mice. However, the overexpression of active form of NRG1t3 in NRG1t3ΔC mice results in the enhanced expression of PMP2 compared to control mice, even in absence of BACE1 expression (*Bace1*^−/−^; NRG1t3ΔC) (Figure 1D). This suggests that PMP2 expression dependents on the level of activation of NRG1t3. More precisely, PMP2 expression is directly regulated by BACE1-dependent activation of NRG1t3.

We determined that the upregulation of PMP2 is not limited to Schwann cells associated with somatic neurons overexpressing NRG1t3 but is also occurring in the autonomic nervous system, as shown by the 3-fold increase in PMP2 relative intensity in the NRG1t3 overexpressing vagus nerve (Figure S1). We then evaluated if *Fabp8* (encoding for PMP2) was the only FABP gene upregulated downstream NRG1t3 overexpression. Four genes coding for fatty acid binding proteins are known to be expressed in the mouse sciatic nerve postnatally (*Fabp4*, *Fabp5*, *Fabp7*, *Fabp8*) (Gerber et al., 2021). Using reverse transcription, we confirmed the expression of *Fabp3*, *Fabp4*, *Fabp5*, *Fabp7 and Fabp8 cDNA* in mouse sciatic nerve at 30 days of age (Figure S2A). Using quantitative PCR, we determined that *Fabp8* (encoding for PMP2) was the only FABP gene being upregulated by the axonal NRG1t3 overexpression in mouse sciatic nerves at 30 days of age (Figure S2B). Also, *Fabp12* cDNA expression was too low to be accurately quantified, with cycle threshold values that was over 30. While PMP2 is a unique myelin protein component and a FABP family member highly up-regulated downstream NRG1t3 overexpression (Belin, Ornaghi, et al., 2019), whether PMP2 is necessary to promote the NRG1t3-mediated hypermyelination phenotype is unknown.

### 3.2 | PMP2 is required for NRG1t3-mediated hyper-remyelination

**FIGURE 2:**
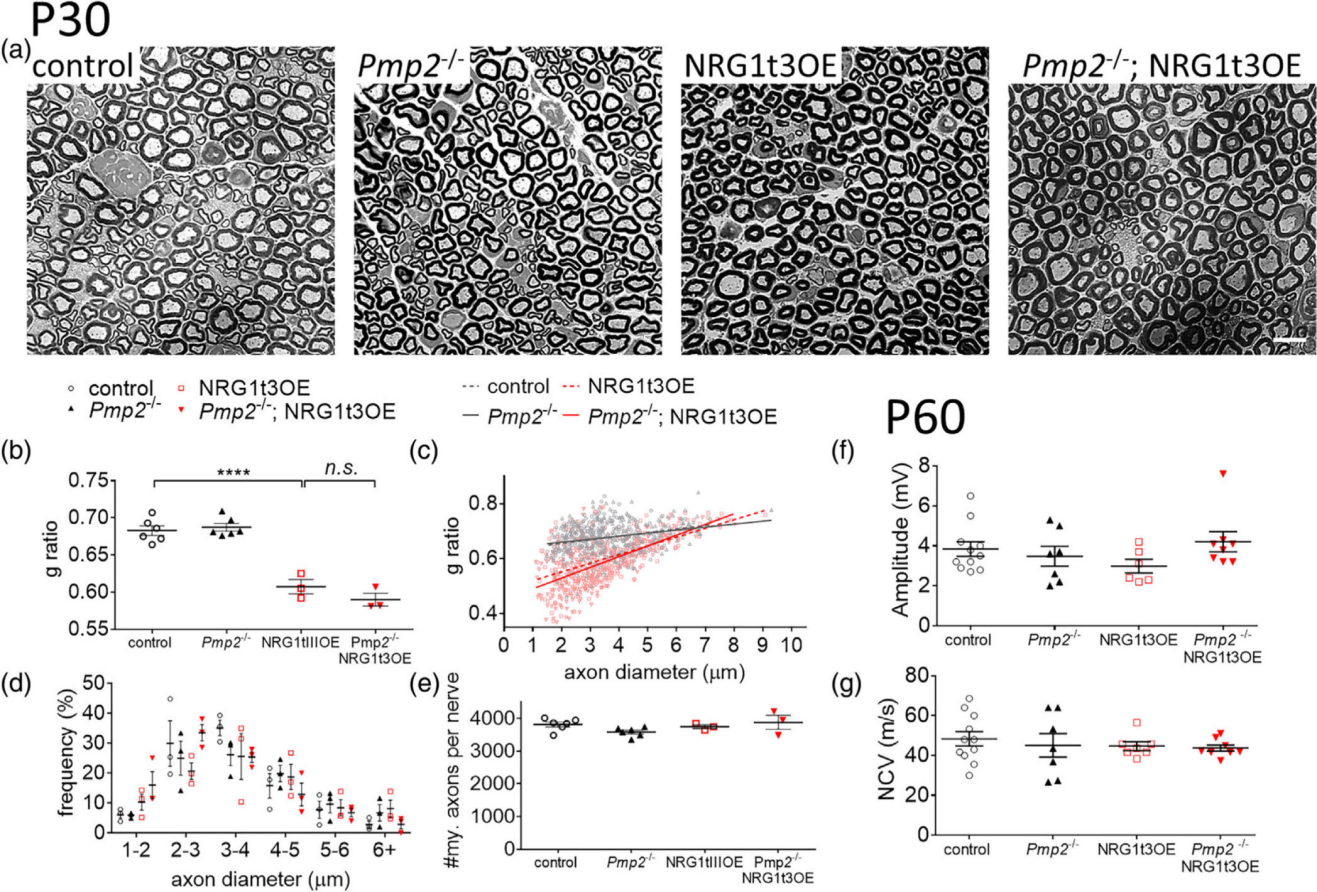
PMP2 is not necessary for NRG1t3-mediated hyper-myelination. (A–E) Semithin analysis of control, *Pmp2*^−/−^, NRG1t3OE, and *Pmp2*^−/−^; NRG1t3OE sciatic nerves at 30 days of age. The thickness of myelin (B,C), the distribution of diameter of myelinated axons (D), and the density of myelinated fibers (E) were measured. (F,G) Measurements of compound muscle action potential (F), and nerve conduction velocity (G) of control, *Pmp2*^−/−^, NRG1t3OE, and *Pmp2*^−/−^; NRG1t3OE mice at 60 days of age. Error bars represent s.e.m, *n* = 3–11 mice, each point on the B, C–F, G graphs represents a different *n*. Each dot on the C graph represents a different myelinated fiber. One-way ANOVA with Bonferroni correction. ****p value <.0001.

The elevation of axonal NRG1t3 expression in NRG1t3 transgenic mice increases myelin thickness both during development (Michailov et al., 2004; Nave & Salzer, 2006; Taveggia et al., 2005) and following nerve injury (Stassart et al., 2013). To clarify the role of PMP2 in NRG1t3-mediated hypermyelination during development and remyelination, we generated mice overexpressing NRG1t3 and knocked out for *Pmp2*. *Pmp2* is encoded by 4 exons and *Pmp2* knockout mice present a 3.1 kb deletion which includes the totality of *Pmp2* coding sequence (i.e., a total deletion of the exons 2 and 3 as well as partial deletion of exons 1 and 4). Morphometric analysis of sciatic nerves at post-natal day 30 (P30) shows that loss of expression for PMP2 does not alter the NRG1t3-mediated hypermyelination in sciatic nerves of *Pmp2*^−/−^;NRG1t3OE mice ( *g* ratio 0.66 ± 0.01) compared to NRG1t3OE mice (*g* ratio 0.65 ± 0.01) (Figure 2A–C). We confirm that the overall distribution of axon diameters is not altered in the different genotypes (Figure 2D). Also, the density of myelinated axons is unchanged (Figure 2E). Measurements of nerve conduction velocity and nerve amplitude from sciatic nerves in NRG1t3OE mice versus *Pmp2*^−/−^; NRG1t3OE mice confirm the absence of functional alteration (Figure 2F,G). Morphometric analysis in early development (P7) and late development (P60) confirm the non-essential function of PMP2 downstream of NRG1t3 overexpression during early and late development (Figure S3). Finally, we observe that while *Pmp2* is ablated and myelin thickness is still increased in *Pmp2*^−/−^;NRG1t3OE compared to *Pmp2*^−/−^ mice sciatic nerves, there is no compensatory expression from genes coding for other fatty acid binding proteins at P30 in sciatic nerves (Figure S2).

**FIGURE 3:**
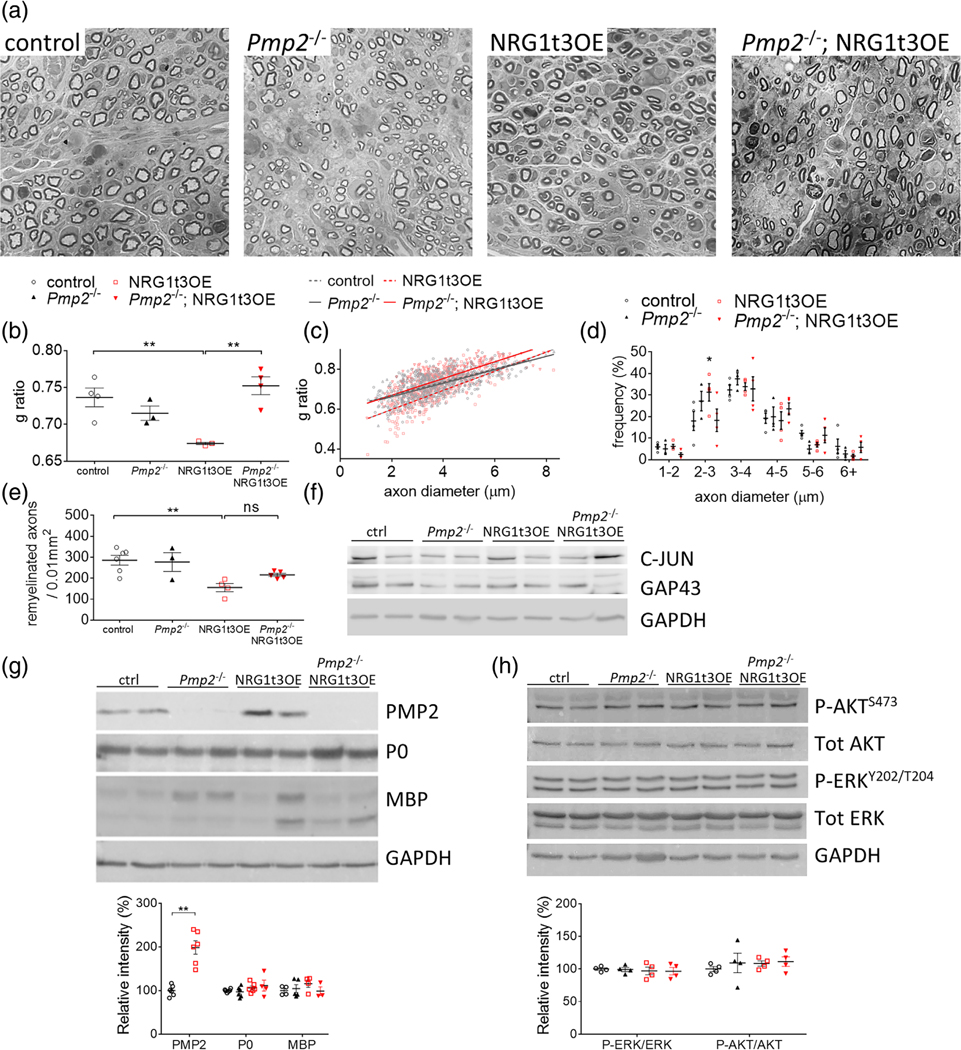
PMP2 is required for NRG1t3-mediated hyper-remyelination. (A–E) Semithin analysis of control, *Pmp2*^−/−^, NRG1t3OE, and *Pmp2*^−/−^; NRG1t3OE crushed sciatic nerves at 20 dpi. The thickness of myelin (B,C), the distribution of diameter of myelinated axons (D), and the density of myelinated fibers (E) were measured. Error bars represent s.e.m, *n* = 3–6 mice, each point on the B, D and E graphs represents a different *n*, and each point on the C graph represents a different myelinated fiber. Scale bars, 10 μm. All images were acquired at the same magnification. One-way ANOVA with Bonferroni correction. (F) Western blot analysis for c-JUN and GAP43 on control, *Pmp2*^−/−^, NRG1t3OE, and *Pmp2*^−/−^; NRG1t3OE sciatic nerves in crushed nerves at 20 dpi. GAPDH was used as a protein loading control. (G) Western blot and densitometry analysis for PMP2, P0, and MBP on control, *Pmp2*^−/−^, NRG1t3OE, and *Pmp2*^−/−^; NRG1t3OE sciatic nerves in crushed nerves at 20 dpi. GAPDH was used as a protein loading control. (H) Western blot for P-AKT Ser473, total AKT, P-ERK 1/2 and total ERK 1/2 and densitometry analyses of P-ERK/tot ERK and P-AKT/tot AKT ratio in control, *Pmp2*^−/−^, NRG1t3OE, and *Pmp2*^−/−^; NRG1t3OE crushed sciatic nerves at 20 dpi. GAPDH was used as a protein loading control. *p value <.05; **p value <.01.

Distinct from unaltered developmental myelination, *Pmp2*^−/−^ mice present transient structural (hypomyelination) and functional changes (reduced nerve conduction velocity) in remyelination following acute nerve crush injury (Stettner et al., 2018). NRG1t3 overexpression also results in Schwann cell hypermyelination during remyelination (Stassart et al., 2013). Thus, we next addressed a possible requirement for PMP2 during NRG1t3-mediated ‘hyper-remyelination’ by assessing myelin thickness and the number of remyelinated axons in crushed sciatic nerves of NRG1t3OE versus *Pmp2*^−/−^;NRG1t3OE compared to *Pmp2*^−/−^ and wildtype mice. At 20 days post-injury (20 dpi), the remyelination in *Pmp2*^−/−^ sciatic nerve (g ratio 0.715 ± 0.017) was not altered when compared to control nerves (g ratio 0.736 ± 0.025) (Figure 3A–C). These results are in accordance with a previous study reporting that loss of *Pmp2* induces remyelination defects at 7 dpi, indicated by a reduced myelin thickness, that is rapidly compensated by 14 dpi (Stettner et al., 2018). We confirmed that NRG1t3 overexpression enhances myelin thickness during remyelination (*g* ratio 0.68 ± 0.01) to the same degree as during development (g ratio 0.65 ± 0.01), including a pronounced hypermyelination of small caliber axons (Figure 3A–C), as previously reported (Stassart et al., 2013). But more importantly, we demonstrate that loss of PMP2 expression significantly reduces myelin thickness in *Pmp2*^−/−^;NRG1t3OE compared to NRG1t3OE during remyelination. The g ratio is significantly increased in *Pmp2*^−/−^; NRG1t3OE (*g* ratio 0.752 ± 0.02) compared to NRG1t3OE (*g* ratio 0.674 ± 0.003) sciatic nerves at 20 dpi (Figure 3A–C). We also confirm that despite a significant punctual increase in the frequency of myelinated axons with a diameter between 2 and 3 μm in NRG1t3OE mice when compared to control mice, the overall distribution of axon diameters is not altered in the different genotypes (Figure 3D). The reduced myelin thickness in sciatic nerve measured in *Pmp2*^−/−^;NRG1t3OE mice and comparable to WT myelin thickness, is persistently reduced at later time point during remyelination (60 dpi) in *Pmp2*^−/−^; NRG1t3OE mice (g ratio 0.71 ± 0.01) compared to NRG1t3OE mice (g ratio 0.69 ± 0.01) (Figure S4A,B).

In NRG1t3OE injured nerves at 20 dpi, PMP2 is upregulated compared to WT (Figure 3G). In addition, we found that the absence of PMP2 expression in the sciatic nerve of *Pmp2*^−/−^; NRG1t3OE mice was not associated with defects in other myelin proteins such as P0, MBP. Similarly c-JUN, the Schwann cell differentiation marker, and GAP43, a marker of axonal regrowth, were unchanged (Figure 3G). Based on our previous work showing that the overexpression of NRG1t3 activates both AKT and ERK downstream pathways (Belin, Ornaghi, et al., 2019; Scapin et al., 2019), we determined if similar regulation downstream the overexpression of NRG1t3 during nerve remyelination exists and if PMP2 up-regulation in NRG1t3OE is required for the activation of AKT and ERK pathway during remyelination. Following sciatic nerve crush injury, at 20 dpi, both AKT and ERK pathways were not dysregulated downstream elevated expression of NRG1t3 (NRG1t3OE) compared to control sciatic nerves (Figure 3H,I). Similarly, the loss of PMP2 expression in *Pmp2*^−/−^ or downstream NRG1t3OE in *Pmp2*^−/−^:NRG1t3OE injured nerves did not affect the activation level of both AKT and ERK pathways compared to control injured nerves. Together these results suggest that elevated expression of NRG1t3 might activate distinct signaling pathways controlling the hyper-remyelination, different from the one activated during development (Belin, Ornaghi, et al., 2019; Scapin et al., 2019). These results also suggest that a PMP2-dependent increase in remyelination in NRG1t3OE does not depend on an increased activation of promyelinating pathways ERK and AKT. At the morphological level, we also observed a significant decrease in remyelinated axon density per unit area at 20 and 60 dpi in NRG1t3OE mice (Figures 3E, S4C) most likely due to an overall reduced fiber density per unit area due to the increase of sciatic nerve size following hypermyelination of the fibers and the increase in extracellular matrix deposition as previously observed in NRG1t3OE mice compared to controls (Scapin et al., 2019). Importantly loss of PMP2 in NRG1t3OE significantly increases the number of remyelinated axons at 60 dpi compared to NRG1t3OE and is comparable to control conditions (Figure S4). This suggests that the overall fiber density is restored following the loss of PMP2-dependent hypermyelination in NRGt13OE injured nerves.

Together, these results demonstrate that boosting remyelination by elevated axonal NRG1t3 expression critically depends on PMP2 functions and indicates a NRG1t3-controlled remyelination program distinct from developmental myelination. However, how upregulation of PMP2 downstream the axonal NRG1t3 overexpression regulates Schwann cells molecular and cellular metabolism during remyelination is unknown.

### 3.3 | PMP2 regulates NRG1t3-mediated fatty acid uptake and ATP production

**FIGURE 4:**
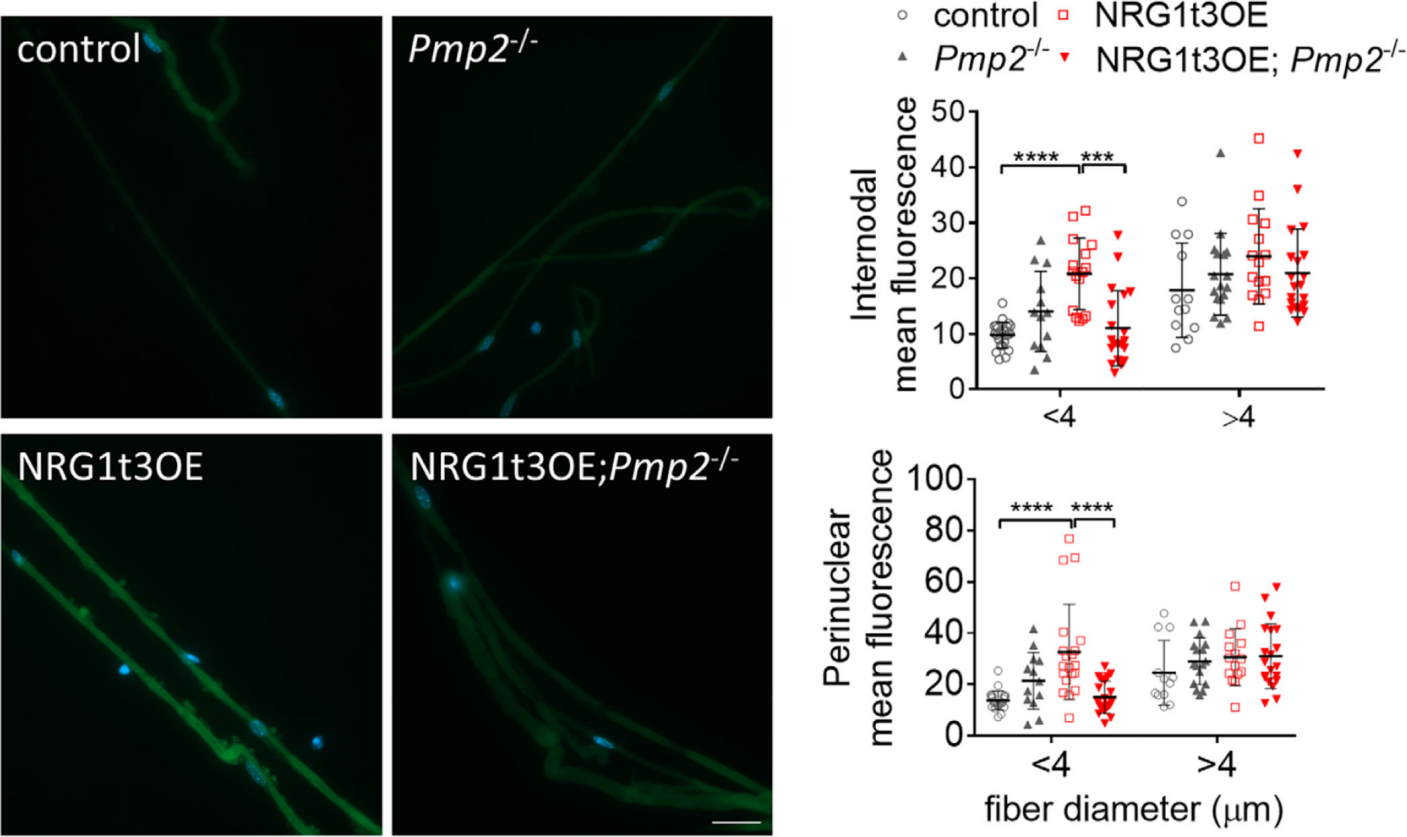
PMP2 is required to promote an increase in fatty acid uptake in sciatic nerve of mice overexpressing NRG1t3. Teased fibers from control, *Pmp2*^−/−^, NRG1t3OE, and *Pmp2*^−/−^; NRG1t3OE sciatic nerves at 20 days of age were incubated for 1 h with C16-labeled BODIPY (10 μM). Intensity of Bodipy was measured in the perinuclear region and in the internodal region. Error bars represent s.d., *n* = 12–25 teased fibers, and each point on the graph represents a different *n*. One-way ANOVA with Bonferroni correction. ****p value <.0001. Scale bar 20 μm.

FABPs are involved in the uptake of extracellular fatty acids in various organs with active lipid metabolism (heart, skeletal muscle, adipose tissue) (Furuhashi & Hotamisligil, 2008; Hotamisligil & Bernlohr, 2015), suggesting that PMP2 could also promote the uptake of fatty acids by Schwann cells and their delivery to specific intracellular compartments (e.g., mitochondria for fatty acid *β* oxidation) (Murphy et al., 1996; Prows et al., 1995). Thus, we next investigated if a NRG1t3OE-mediated increase in PMP2 expression in Schwann cells affects the uptake of extracellular fatty acids. We applied fluorescently labeled palmitic acid (BODIPY-C16) ex-vivo onto teased sciatic nerve fibers isolated from WT, *Pmp2*^−/−^, NRG1t3OE, and *Pmp2*^−/−^; NRG1t3OE mice at 15 days of age, a developmental time point with highly active myelination and active lipid metabolism. We show that compared to WT, the axonal overexpression of NRG1t3 increases the uptake of BODIPY by 113% in the internodal and perinuclear areas of teased sciatic fibers smaller than 4 μm (Figure 4). While lack of PMP2 in *Pmp2*^−/−^ mutants has no impact on the uptake of fatty acid in sciatic nerve fibers compared to wildtype, the loss of PMP2 downstream of NRG1t3 overexpression reduces fatty acid uptake by 47% both in internodal and perinuclear of teased sciatic fibers smaller than 4 μm (Figure 4). Thus, the increased expression level of PMP2 in Schwann cells, known to be up-regulated at 70% in mice overexpressing NRG1t3 (Belin, Ornaghi, et al., 2019; Scapin et al., 2019), increases the uptake of extracellular fatty acid in nerve fibers especially in fibers of small calibers (<4 μm) which are known to be highly hypermyelinated by NRG1t3 overexpression (Figure 2C) (Michailov et al., 2004; Nave & Salzer, 2006; Taveggia et al., 2005).

**FIGURE 5:**
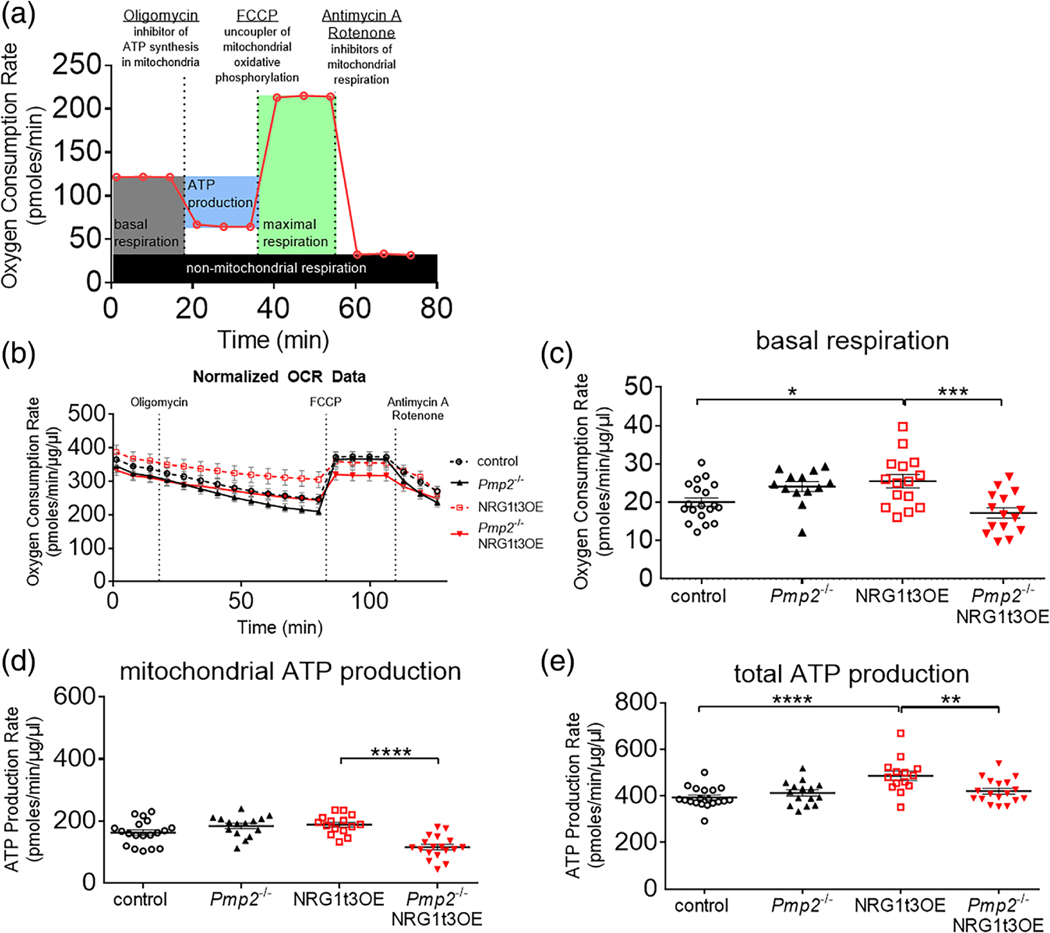
PMP2 regulates NRG1t3-mediated ATP production and basal respiration during remyelination. (A) Schematic representation of the Mitochondrial stress test. (B) Oxygen consumption rate in control, *Pmp2*^−/−^, NRG1t3OE, and *Pmp2*^−/−^; NRG1t3OE in crushed sciatic nerves at 7 dpi. Recordings were taken every 393 s. Oligomycin was injected at 15 min, FCCP at 81 min, and Antimycin A/Rotenone at 107 min. The last recording was taken at 126 min. (C–E) basal respiration (C), mitochondrial ATP production (D), and total ATP production (E) were extrapolated from the OCR and ECAR measurements in control, *Pmp2*^−/−^, NRG1t3OE, and *Pmp2*^−/−^; NRG1t3OE in crushed sciatic nerves at 7 dpi. Error bars represent s.e.m, *n* = 13–19 nerves, and each point on the graph represents a different n. One-way ANOVA with Bonferroni correction. *p value <.05; **p value <.01; ***p value <.001; ****p value <.0001.

Once fatty acids enter the cells, FABPs may actively facilitate their transport to specific cellular compartments, such as mitochondria for *β* oxidation and ATP synthesis. Thus, we measured the consequence of *Pmp2* ablation in NRG1t3 overexpressing mice by measuring OCR and ECAR from sciatic nerve segments. When we measured oxygen consumption in sciatic nerve at P30 before and after treatment with oligomycin, an inhibitor of ATP synthesis in mitochondria (Figure 5A), we found that loss of PMP2 reduced mitochondrial ATP production both in *Pmp2*^−/−^ and *Pmp2*^−/−^;NRG1t3OE (Figure S5A,D), while there was no significant difference for basal, maximal respiration and total ATP production between in *Pmp2*^−/−^ and *Pmp2*^−/−^; NRG1t3OE (Figure S5A–C). Interestingly, concomitantly to the reduction in mitochondrial ATP production, loss of PMP2 in *Pmp2*^−/−^; NRG1t3OE was associated with increased glycolytic ATP production (Figure S5E). This suggests that in a highly metabolic demanding condition, such as during NRG1t3-mediated hypermyelination and in absence of Schwann cell fatty acid chaperone (PMP2), Schwann cells adapt their metabolic function favoring glycolysis to ensure stable ATP production. Since the glycolytic shift in Schwann cells was described during injury (Babetto et al., 2020), we conducted similar measures on injured nerves. Following nerve crush injury, during early remyelination (7 dpi) in sciatic nerves, loss of PMP2 downstream NRG1t3 overexpression significantly decreases the basal respiration (−33%), mitochondrial ATP production (−39%) and total ATP production (−14%) in *Pmp2*^−/−^;NRG1t3OE versus NRG1t3OE mice (Figure 5C–E). No difference between genotypes was observed regarding maximal respiration and glycolytic ATP production (Figure S5G,H). However, during remyelination, loss of PMP2 in *Pmp2*^−/−^; NRG1t3OE does not lead to an increase of glycolytic ATP, and the total ATP production is reduced by 14%. This suggests that in a highly metabolic demanding condition, such as during early remyelination and the NRG1t3OE-mediated hypermyelination, Schwann cells cannot adapt their metabolic function in the absence of PMP2 to ensure stable ATP production. In addition, the absence of increased mitochondrial ATP production seen in NRG1t3OE (Figure S5) suggests that the increase in fatty acid uptake we observed during development (Figure 4) is perhaps preferentially used in lipid synthesis to support the hypermyelination program. Overall, these results suggest new roles for PMP2 downstream of NRG1t3 in regulating Schwann cell fatty acid uptake and energy homeostasis. It also suggests that fatty acid uptake and/or mitochondrial ATP production may be associated with hypermyelination downstream NRG1t3 during remyelination, but further studies will be necessary to carefully evaluate how much these metabolic regulations mediated by PMP2 contribute to the regulation of myelination and remyelination in Schwann cells.

## 4 | DISCUSSION

While it is well known that axonal NRG1t3 regulates myelin formation through EGR2-dependent gene expression and lipid synthesis, we previously found that enhancing axonal NRG1tIII signaling increases fatty acid levels in myelin, improves myelination and nerve function in mouse models for inherited demyelinating neuropathy but was not coupled to the activation of the key myelin gene transcription factor EGR2 nor by a global myelin protein up-regulation (Belin, Ornaghi, et al., 2019; Scapin et al., 2019). Instead, we found that rather than a global myelin protein up-regulation, PMP2, a fatty acid chaperone, is uniquely up-regulated downstream NRG1t3 overexpression in Schwann cells.

Here we investigate whether *Pmp2* expression in Schwann cells was necessary to mediate NRG1t3 promyelinating functions. Using mice overexpressing axonal NRG1t3 and *Pmp2* knocked out (*Pmp2*^−/−^;NRG1tIIIOE), we demonstrate that PMP2 is necessary for improving NRG1t3-mediated remyelination, through hypermyelination, after nerve crush injury. We also show that PMP2 is required to promote an increase in fatty acid uptake in sciatic nerve fibers of NRG1t3OE mice. Interestingly, one study has reported that ectopic expression of PMP2 in astrocytes leads to an increase in both cell volume and the number of processes produced by astrocytes (Kelley et al., 2018). Thus, one could entertain that it is possible that this phenotype observed in NRG1t3OE results from the activation of growth pathways, such as PTEN, ERK, AKT or mTORC, which have all been shown to regulate myelin thickness; and that PMP2 somewhat contributes to the regulation of this pathway. Our results, however, show that both ERK and AKT pathways are not being dysregulated when PMP2 is upregulated or absent during early remyelination following sciatic nerve crush. Finally, we show that while the overexpression of axonal NRG1t3 does not increase ATP production in Schwann cells during remyelination, the increased amount of fatty acid uptake in NRG1t3OE, PMP2 could support the fatty acid utilization for lipid synthesis during remyelination. However, we show that loss of *Pmp2* expression in *Pmp2*^−/−^;NRG1tIIIOE sciatic nerves reduces mitochondrial ATP production and total ATP production. This suggests that during remyelination Schwann cells rely on PMP2 expression and possibly on sustained ATP production during high metabolic demands such as during remyelination and NRG1t3-mediated hypermyelination. Also, it suggests that while during development Schwann cells can compensate for the reduction in mitochondrial ATP production mediated by the loss of PMP2 expression by increasing glycolytic ATP production, during repair, Schwann cell knock-down for PMP2 cannot assume this metabolic shift which led to lower amount of ATP production. In sum, our data suggests that prolonged high expression of PMP2 is necessary for enhancing remyelination in Schwann cells and that PMP2 up regulation could help modulate the utilization of fatty acid to properly respond to energetic demand during nerve repair.

De novo fatty acid synthesis is critical for the correct formation and growth of myelin both in the PNS and in the CNS (Camargo et al., 2017; Cermenati et al., 2015; Dimas et al., 2019; Montani et al., 2018; Verheijen et al., 2009). Besides fatty acid synthesis, myelinating cells have the ability to uptake fatty acids (Poitelon et al., 2020), and rely, to a certain extent, on fatty acid uptake for myelin biosynthesis (Hinder et al., 2014; Huey et al., 1998; Obrosova et al., 2007). We show here that in NRG1t3OE mice, which produce more myelin, fatty acid uptake is enhanced in small fibers in sciatic nerve, and that this increase of fatty acid uptake is mediated by PMP2/FABP8 expressed in Schwann cells. In addition, NRG1t3OE mice overexpress Nrg1 type III under the control of neuronal promoter Thy1.2, targeting mainly sensory axons. Thus, it is not surprising that our data indicates that the effect of NRG1t3OE on myelin thickness or fatty acid uptake predominantly affect nerve fibers of small caliber. Fatty acid uptake occurs through passive diffusion or active recruitment by fatty acid translocase and fatty acid transport proteins and is facilitated by FABPs (Murphy, 2017; Murphy et al., 1996; Prows et al., 1995). Notably, FABPs can regulate the extracellular fatty acid uptake in cells, by interacting with fatty acid transporters (Eto et al., 2003; Kalinowska et al., 2009; Spitsberg et al., 1995), transfer lipids from/to membranes via collisional interaction with phospholipid-rich membrane to release or accept fatty acid from donor membrane (Abe et al., 2021; Zenker et al., 2014) and modulate lipid synthesis rate and lipid composition in most cells (Hotamisligil & Bernlohr, 2015; Murphy et al., 2000; Veerkamp & van Moerkerk, 1993; Woodford et al., 1993). Fatty acid uptake was proposed to compensate partially for deficiencies in fatty acid synthesis in both the CNS and the PNS (Dimas et al., 2019; Montani et al., 2018), and more evidently in myelinating cells in direct proximity to blood vessels or indirectly from an increase horizontal flux of fatty acids through astrocytes or adipocytes (Camargo et al., 2017; Montani et al., 2018). While in other cellular systems, several mechanisms have been proposed to support the role of FABPs in fatty acid uptake, future work will be necessary to understand by which of these mechanisms PMP2 regulates fatty acid uptake in nerve fiber and potentially Schwann cells. Interestingly, while it was previously described that PMP2 is only expressed in a subpopulation of myelinating Schwann cells that form thicker myelin (Trapp et al., 1979; Trapp et al., 1984; Zenker et al., 2014), recent work has shown that the subpopulation of Schwann cells expressing PMP2 is predominantly myelinating motor axons (Yim et al., 2022). Thus, it is tempting to think that the overexpression of axonal NRG1t3, hypermyelination small caliber axons, affects more importantly Schwann cells that were not naturally expressing PMP2 (i.e., Schwann cell myelinating sensory neurons), but this remains to be explored.

Fatty acids can be used for the synthesis of complex lipids which can then be used to form myelin sheets, but they can also be used for fatty acid oxidation. Because myelinating cells need to produce high levels of complex lipids for myelin formation, there is a long-standing belief that myelinating cell metabolism does not favor fatty acid oxidation (Schonfeld & Reiser, 2013). Yet it is possible for myelinating cells to adapt their fatty acid utilization for lipid synthesis to ATP production. For example, mice lacking SREBF1 (a key transcription factor in lipid homeostasis) manifest a decrease in fatty acid synthesis and an increase in fatty acid *β* oxidation, and yet despite this, these mice demonstrate an increase in myelin thickness (Cermenati et al., 2015). Our data show that increased remyelination seen in NRG1t3OE mice is correlated with an increase in mitochondrial ATP production. While it is still unclear through which mechanisms mitochondrial ATP production occurs in NRG1t3OE sciatic nerves, one could postulate that the upregulation of PMP2 may favor fatty acid *β* oxidation. Indeed FABPs can carry and exchange intracellular fatty acids to specific intracellular compartments in which fatty acids play a modulatory role (e.g., endoplasmic reticulum for membrane synthesis, mitochondria for fatty acid *β* oxidation, nucleus for lipid-mediated transcriptional regulation) (Murphy, 2017; Murphy et al., 1996; Prows et al., 1995). In addition, no studies have investigated specifically whether fatty acid *β* oxidation is required to accommodate the energetic requirement of myelinating cells and if it is coupled to myelin formation. Notably, Viader et al. reported that mitochondrial dysfunction in Schwann cells causes demyelination and axonal degeneration. This phenotype is presumably caused by the toxic accumulation of acylcarnitine, the disruption of the integrated stress response, and through an increase in fatty acid *β* oxidation (Viader et al., 2013). Thus, whether increase in mitochondrial ATP production and fatty acid *β* oxidation is beneficial or detrimental to myelin production is still unclear. Apart from mitochondria, peroxisomes which are abundant organelles in the myelin are also involved in fatty acid *β* oxidation. It will be important to study how PMP2, as a fatty acid chaperone, regulates peroxisomal *β* oxidation in the myelinating Schwann cells. Peroxisomes are responsible for the *β* oxidation of very long-chain fatty acids, where the resulting fatty acids are preferentially transported by FABPs and could serve as metabolic support to axons (Kassmann, 2014). In addition, peroxisomes have been demonstrated to reside in non-compact myelin channels and are important for the long-term integrity of the myelin (Richert et al., 2014).

While the role of PMP2 was previously investigated in loss of function in vivo models, the potential beneficial gain of function of PMP2 upregulation, as our previous studies suggested (Belin, Ornaghi, et al., 2019; Scapin et al., 2019), was unexplored. While *Pmp2* is only expressed in rodent Schwann cells, in humans, *PMP2* is also expressed in central nervous system astrocytes and oligodendrocytes (Zhang et al., 2014; Zhang et al., 2016). Interestingly ectopic expression of PMP2 in mouse astrocytes causes them to increase in size and to more closely resemble their human counterparts (Kelley et al., 2018). Thus, investigating the potential role of PMP2 in the central nervous system is of great interest as well as it could be associated with evolutionary advantages in other glial cells.

In addition, despite that ablation of PMP2 alone does not seem to have a discernable role in Schwann cell biology during myelination or remyelination, our data demonstrate that PMP2 is required during remyelination in mice overexpressing NRG1t3. It is possible that in demyelinating conditions, such as CMT1G, there is a greater reliance on Neuregulin 1 type III for peripheral nerve myelin maintenance and/or remyelination. Indeed, there is a growing body of work suggesting that dysregulation of the NRG1 type III/ERBB2/3 signaling in Schwann cells isa common pathogenic mechanism for several demyelinating CMT subtypes (El-Bazzal et al., 2023; Jiang et al., 2022; Lee et al., 2017). Thus, the causal pathomechanism behind PMP2 mutations in CMT1G might be dysregulation of the NRG1t3 signaling pathway. However, it is important to note that the disruption of the NRG1t3 signaling pathway is not a universal pathomechanism to demyelinating neuropathies (e.g., CMT1B and CHN shown no dysregulation of the NRG1t3/ERBB2/3 signaling (Belin, Ornaghi, et al., 2019; Scapin et al., 2019)).

In sum, our data show that PMP2 is necessary for NRG1t3OE-mediated remyelination. However, because the absence of PMP2 does not affect NRG1t3OE-mediated myelination during development, it indicates the role of PMP2 in the regulation of myelination is complex. First, while there are many studies supporting the difference between myelination and remyelination, the difference between these two biological processes is still not well understood. Given our results, it is possible that in contrast to developmental myelination, increased remyelination in NRG1t3OE mice depends on metabolic support functions provided by NRG1t3-PMP2 axis. Second, there are now several studies showing that PMP2 is a marker for a specific subtype of Schwann cells, but the characteristics of this subpopulation of Schwann cells and how they are regulated especially during demyelination and remyelination remains unknown.

## Supplementary Material

Table S1

Supinfo 1

## Data Availability

The data that support the findings of this study are available from the corresponding author upon reasonable request.
