# Supplementary material for: PMP2 regulates myelin thickening and ATP production during remyelination
Source: Glia. Author manuscript; Available in PMC 2025 May 1. (PMC11027087; doi:10.1002/glia.24508)
Supplement: Supinfo 1 [file NIHMS1961171-supplement-Supinfo_1.docx]

**
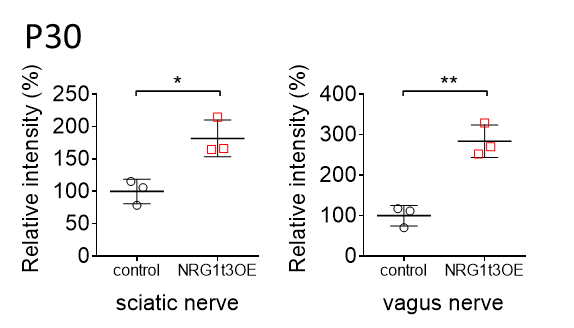

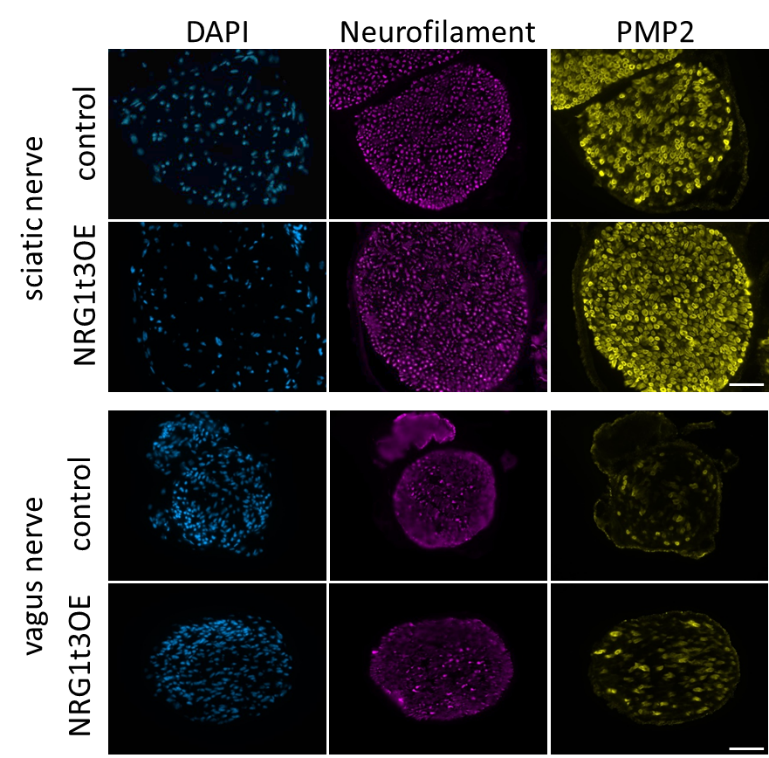
**

**Supplementary Figure 1.** **PMP2 levels in Schwann cells are increased in both autonomic and somatic nerves of mice overexpressing NRG1t3.** (above) Mean fluorescence for PMP2 in cross sections of sciatic and vagus nerves of control and NRG1t3OE animals at 30 days of age. Error bars represent s.e.m., n=3 animals, and each point on the graph represents a different n. Two-tailed unpaired Student's *t*-test. (below) Immunostaining for Neurofilament, PMP2, and DAPI staining in cross-sections of sciatic and vagus nerves of control and NRG1t3OE animals at 30 days of age. Bar = 40µm. * *P*-value <.05, ** *P*-value <.01.

**
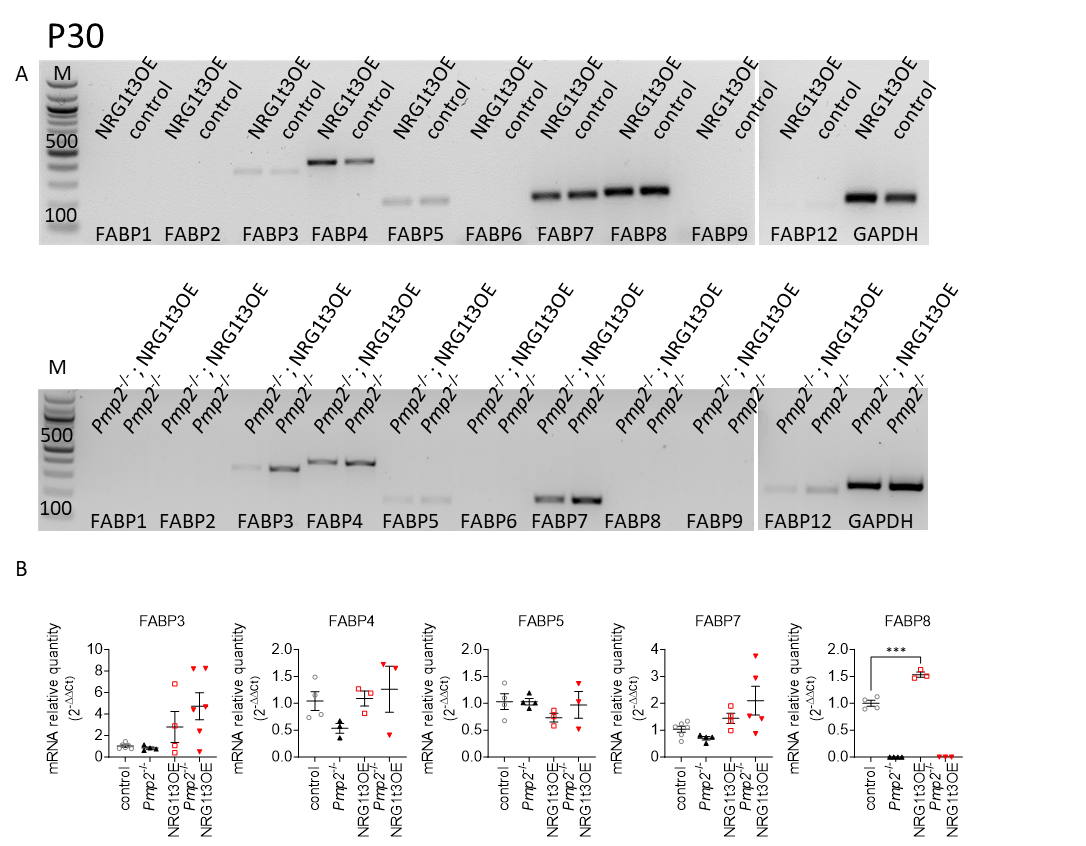
**

**Supplementary Figure 2.** **PMP2 is the only FABP upregulated in mice overexpressing NRG1t3.** (**A**) Polymerase chain reaction (PCR) and (**B**) real-time quantitative PCR (RTq-PCR) analysis of FABPs in control (WT), *Pmp2*^-/-^, NRG1t3OE and *Pmp2*^-/-^ ; NRG1t3OE sciatic nerves at 30 days of age. The genes in orange FABP3, FABP4, FABP5, FABP7, PMP2 (FABP8) were the only FABPs investigated by RTqPCR. *Gapdh* was used as a control for the PCR. *Rps20* was used for the endogenous control for the RTq-PCR. Error bars represent s.e.m, n=3-5 animals, and each point on the graph represents a different n. Two-tailed unpaired Student's *t*-test. * *P*-value <.05.

**
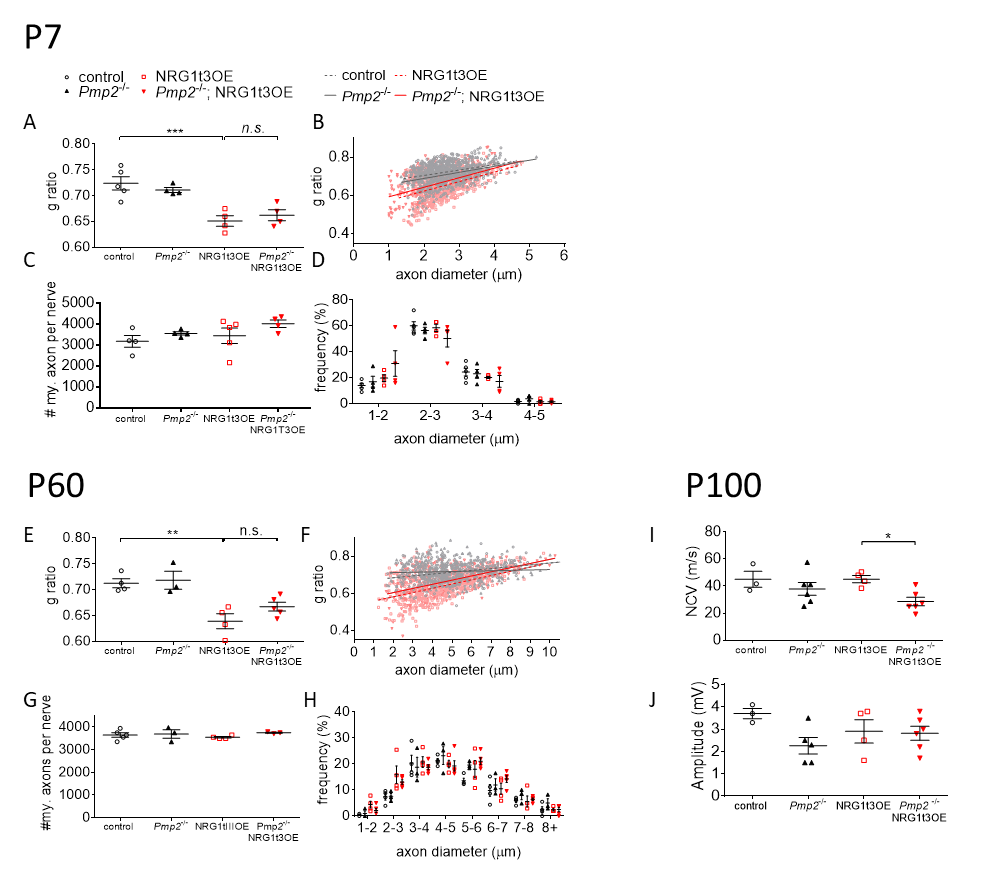
Supplementary Figure 3.** **PMP2 is not necessary for NRG1t3OE-mediated hyper- myelination.** (**A-H**) Semithin analysis of control, *Pmp2*^-/-^, NRG1t3OE, and *Pmp2*^-/-^; NRG1t3OE sciatic nerves at 7 days (**A-D**) and 60 days (**E-H**) of age. The thickness of myelin (**A-B, E-F**), and the number of myelinated fibers (**C-D, G-H**) were measured. Error bars represent s.e.m, n=3-6 animals, each point on the A, C-E, G-J graphs represents a different n. each point on the B and F graph represents a different myelinated fiber. One-way ANOVA with Bonferroni

**
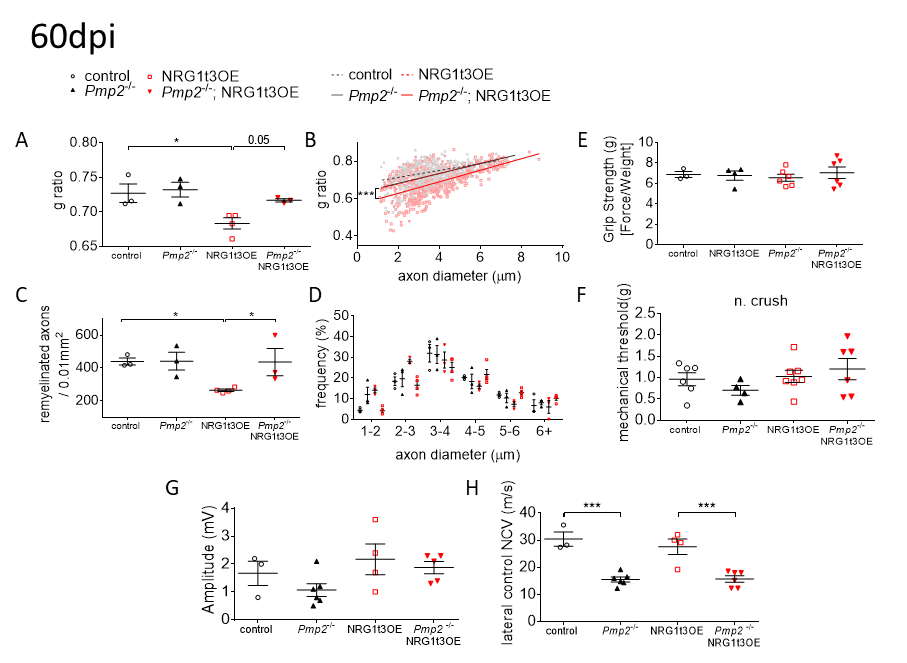
**

**Supplementary Figure 4.** **PMP2 is required for NRG1t3 to mediate hyper-remyelination.** (**A-D**) Semithin analysis of control, *Pmp2*^-/-^, NRG1t3OE, and *Pmp2*^-/-^; NRG1t3OE crushed sciatic nerves at 60 dpi. The thickness of myelin (**A-B**), and the number of myelinated fibers (**C-D**) were measured.  (**E**) Measurements of grip strength of *Pmp2*^-/-^, NRG1t3OE, and *Pmp2*^-/-^; NRG1t3OE animal, 60 days following nerve crush injury. Grip strength measurements were normalized to the weight of the mice. (**F**) Mechanical threshold measurements of *Pmp2*^-/-^, NRG1t3OE, and *Pmp2*^-/-^; NRG1t3OE animal, 60 days following nerve crush injury. Error bars represent s.e.m, n=3-7 animals, each point on the A, C-F graphs represents a different n. each point on the B graph represents a different myelinated fiber. (**G-H**) Measurements of compound muscle action potential (**G**), and nerve conduction velocity (**H**) of control, *Pmp2*^-/-^, NRG1t3OE, and *Pmp2*^-/-^; NRG1t3OE animals at 60 dpi. One-way ANOVA with Bonferroni correction. * *P*-value <.05, *** *P*-value <.001.

**
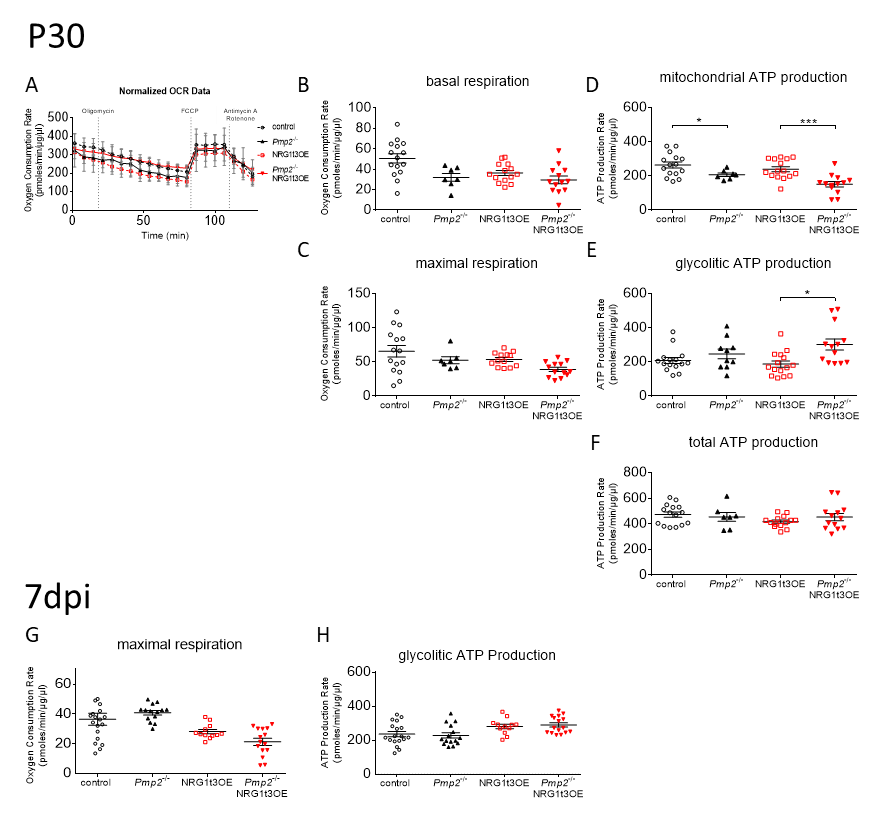
**

glycolytic ATP production

glycolytic ATP production

**Supplementary Figure 5. Loss of PMP2 in mice overexpressing NRG1t3 leads to a shift ATP production**. (**A**) Oxygen consumption rate in control, *Pmp2*^-/-^, NRG1t3OE, and *Pmp2*^-/-^; NRG1t3OE in sciatic nerves at 30 days of age. Recordings were taken every 393s. Oligomycin was injected at 15min, FCCP at 81min, and Antimycin A/Rotenone at 107min. The last recording was taken at 126min. (**B-F**) basal respiration (**B**), maximal respiration (**C**), mitochondrial ATP production (**D**), glycolytic ATP production (**E**), and total ATP production (**F**) were extrapolated from the OCR and ECAR measurements in control, *Pmp2*^-/-^, NRG1t3OE, and *Pmp2*^-/-^; NRG1t3OE in sciatic nerves at 30 days of age. (**G-H**) Maximal Respiration (**G**), glycolytic ATP production (**H**) were extrapolated from the OCR and ECAR measurements in control, *Pmp2*^-/-^, NRG1t3OE, and *Pmp2*^-/-^; NRG1t3OE in crushed sciatic nerves at 7 dpi. Error bars represent s.e.m, n=7-19 nerves, and each dot on the graph represents a different n. One-way ANOVA with Bonferroni correction. **P*-value <.05, *** *P*-value <.001.
